# Consistency and grade prediction of intracranial meningiomas based on fractal geometry analysis

**DOI:** 10.1007/s10143-025-03737-1

**Published:** 2025-08-14

**Authors:** Balázs Markia, Tamás Mezei, János Báskay, Péter Pollner, Adrienn Mátyás, Ákos Simon, Péter Várallyay, Péter Banczerowski, Loránd Erőss

**Affiliations:** 1 Clinic of Neurosurgery and Neurointervention, Semmelweis University, Budapest, Hungary; 2 Department of Neurosurgery, Semmelweis University, Budapest, Hungary; 3 Data-Driven Health Division of National Laboratory for Health Security, Health Services Management Training Centre, Semmelweis University, Budapest, Hungary; 4 Department of Biological Physics, Eötvös Loránd University, Budapest, Hungary

**Keywords:** Fractal geometry analysis, Radiomics, Meningioma, Tumor consistency, Histology, Prediction

## Abstract

Meningiomas are the most common primary tumors in the central nervous system. Surgical resection remains the main treatment option, often resulting in a curative outcome; however, careful preoperative planning is essential. One of the primary concerns for neurosurgeons treating meningiomas is tumor consistency, as this has a significantly impact on the likelihood of complete resection. Predicting the consistency and histology of a meningioma prior to surgery is valuable for selecting the appropriate surgical instruments and planning the approach. We conducted a retrospective study to analyze clinical data and preoperative MRI images of patients who underwent surgery for intracranial meningiomas. T1, T1c, T2, and FLAIR sequences were obtained for all patients. Surgical notes were reviewed to assess tumor consistency. Tumor segmentation was performed using ITK-SNAP software. Fractal analysis and statistical analyses were made, including t-tests, Fisher’s exact tests, logistic regression, and ROC analysis. Forty-eight patients met the selection criteria. For prediction of consistency when only fractal parameters were used, lacunarity index was able to discriminate between soft and hard consistency with an AUC value of 0.745 (95% CI: 0.538–0.958). When tumor homogeneity was added, these values changed to 0.763 (95% CI: 0.518–1.000). For prediction of histological grade, an AUC value of 0.697 (95% CI: 0.490–0.952) was found, using only fractal dimension. When age, tumor homogeneity and volume parameters were added, this value increased to 0.841 (95% CI: 0.625–1.000). Our study suggests that fractal metrics are useful tools for preoperative estimation of tumor consistency and histological grading.

**Supplementary Information:**

The online version contains supplementary material available at 10.1007/s10143-025-03737-1.

## Introduction

Meningioma is the most common primary tumor type of the central nervous system, accounting for one-third of all intracranial tumors [1]. The histological classification of these tumors is divided into three categories: CNS WHO grade 1, 2 and 3. Surgical resection remains the primary treatment option, often leading to a curative outcome for symptomatic patients. The surgical treatment of meningiomas, especially when the tumor is located in areas challenging to access, careful preoperative planning is crucial. One of the key concerns for neurosurgeons dealing with meningiomas is tumor consistency, as this has a significant impact on the likelihood of a complete resection, along with factors such as histological subtype, location, size, and proximity to critical anatomical structures. Preoperative prediction of meningioma consistency is useful for selecting appropriate surgical instruments and planning the surgical approach. It also helps to estimate the difficulty of the procedure, the potential for complete resection, and the therapeutic outcome. Analysis of preoperative MRI imaging characteristics has also shown promising results in estimating histological type and consistency [2].

Fractal geometric analysis of MRI studies were previously shown to provide valuable information on a number of markers such as histological types of breast, prostate cancer, identification of certain subtypes of dementias and brain tumors [3–5]. However, to date, no research has investigated meningioma consistency with the latest methods of fractal geometric analysis of preoperative MRI images.

The human body, along with its organs and tissues, possesses intricate geometric structures, including abnormal tissues and tumors. Characterizing these structures using the metrics of classical Euclidean geometry is challenging and often leads to inaccuracies. The complex geometry of tumors can be described through their shape properties using various measures. Fractal dimension (FD), a concept introduced by Benoit Mandelbrot in 1977 and 1983 [6, 7], is crucial for characterizing irregular and rough shapes. It is a fractional value that reveals the structural complexity of the shape of an object. The lacunarity index (LI) provides insight into the distribution and density of material within the volume of a shape. These parameters can be compared between different patient groups, as well as between unhealthy and healthy controls [8].

The aim of our research was to identify a novel MRI biomarker by fractal geometry analysis that may improve the estimation of preoperative histological grading and tumor consistency to help preoperative surgical planning, maximize resection and minimize morbidity in patients with intracranial meningioma.

## Methods

### Patient selection

A single-center retrospective clinical study of the clinical, radiological and histopathological characteristics of intracranial convexity-parasagittal-falcotentorial meningioma patients who underwent surgery at our Department was conducted. The study was approved by the Local Ethics Committee (IKEB Registration Number: 4/2023) and by the Scientific and Research Ethics Committee of the Hungarian Medical Research Council (ETT-TUKEB Registration Number: BM/26596-1/2023).

We collected data from January 2018 to January 2023. The main inclusion criterion was the availability of the complete DICOM sequence of the preoperative MRI scan (T1, T1c, T2 and FLAIR sequences were mandatory).

#### The primary inclusion criteria included

- Complete DICOM sequence of the preoperative MRI scan (mandatory sequences: T1, T1c, T2, and FLAIR).
- Availability of operative notes with consistency grading.

#### The following pre-, intra- and postoperative clinical parameters were collected

- Age and sex.
- Presenting symptoms (e.g., headache, epileptic seizure, paresis, aphasia, incidental finding, etc.)
- MRI findings: localization (e.g., frontal, temporal, cerebellar, etc.), presence of necrosis or cystic component, quality of contrast enhancement (homogeneous or heterogeneous), and tumor volume.
- Type of surgical resection (e.g., total, subtotal).
- Neuropathological (2021 CNS WHO) diagnosis and features (e.g., grade, presence of necrosis, brain invasion property, TERT promoter mutation, Ki-67 index, etc.)

#### Measuring tumor consistency

Data on tumor consistency were extracted from operative notes, with patients divided into three groups based on the report of the surgeon:

- Soft/suckable with normal suction.
- Hard/non-suckable with normal suction - requiring CUSA or other extra technical measures.
- Miscellaneous.

### Preprocessing of MRI sequences

**Fig. 1:**
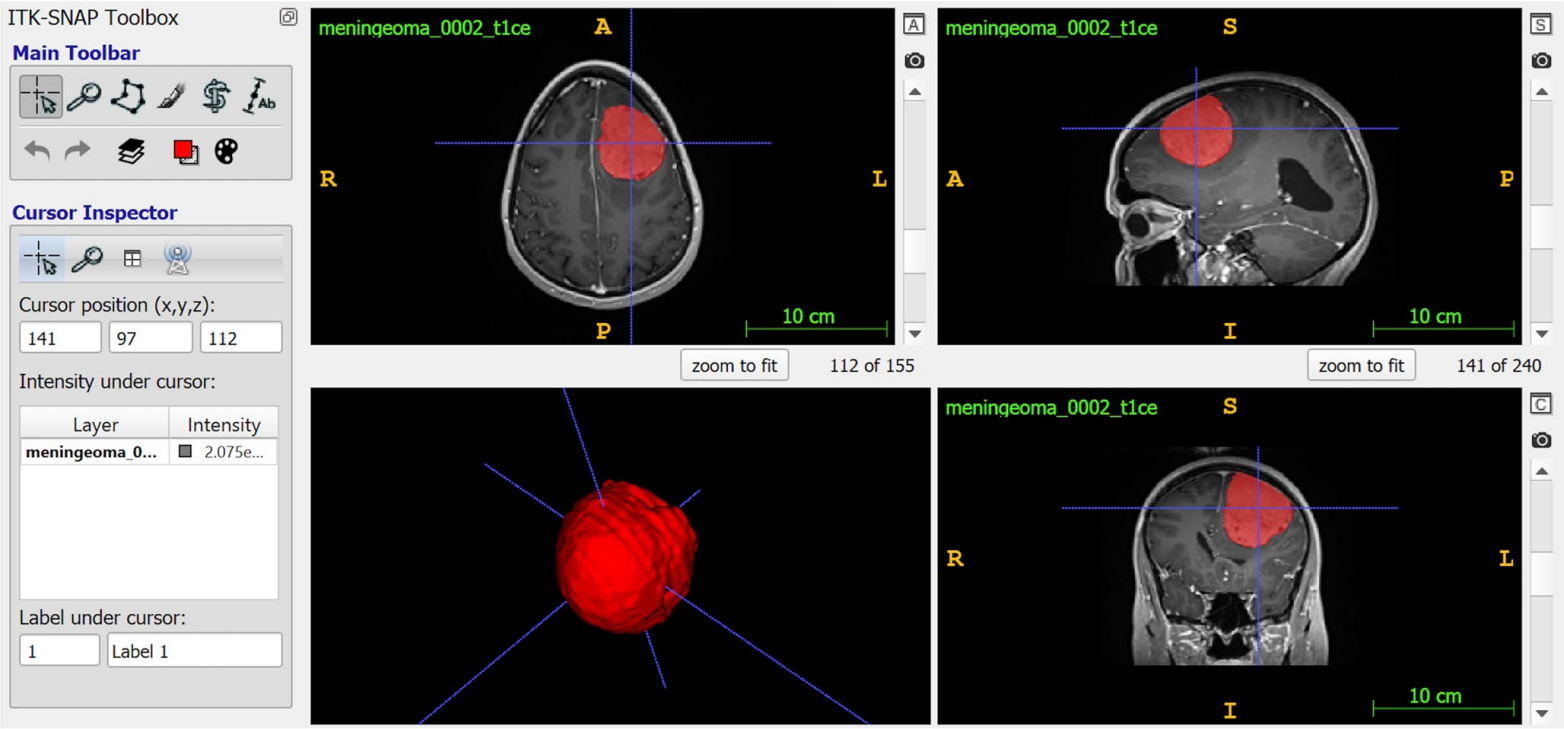
The process of segmentation and fractal analysis. We used ITK-SNAP software for the segmentation of tumors. We made annotations on the axial view using polygon mode for every 3–5 slices and then interpolated to the rest of the image using software. Finally, the program created the spatial model of the tumor on which the fractal analyses were run

MRI sequences were sourced from multiple institutions and acquired using various scanners, resulting in a dataset with a resolution ranging from 0.3 mm³ to 1 mm³ per voxel. To standardize the data, we transformed all sequences to a uniform resolution of 1 mm³ and coregistered them to the T1c sequence using the BraTS Pipeline of the Cancer Imaging Phenomics Toolkit [9, 10]. Tumor segmentation masks were manually annotated by neurosurgeons using ITK-SNAP (version 3.8.0, PICSL, Philadelphia, USA) (Fig. 1).

### Fractal analysis

**Fig. 2:**
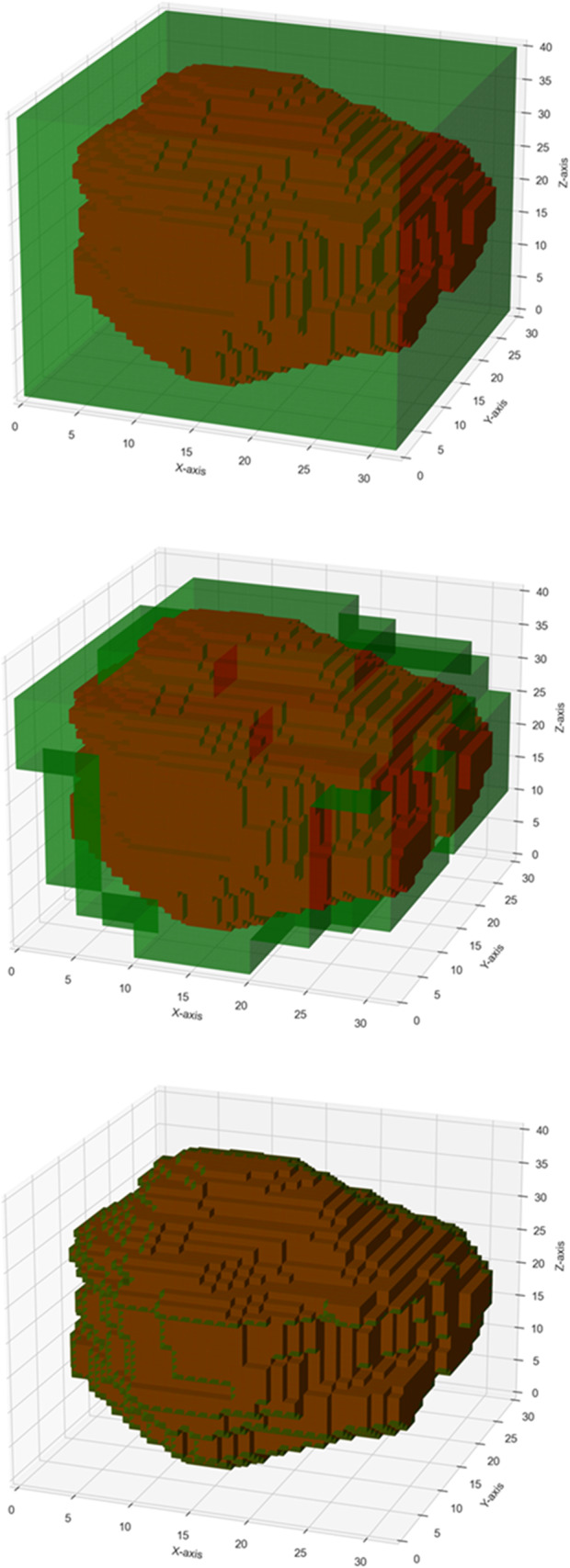
Flowchart showing the box counting methodology used in the fractal analysis

Fractal analysis was performed using an in-house Python tool, “FracND”, which was designed with CPU-parallelized sliding window capability for box counting and lacunarity calculation in any dimensions. The fractal dimension was extracted from the 3D binary segmentation masks by determining the number of non-empty windows (‘box count’) at each given scale and calculating the slope of the log-log plot between the box count and inverted window size (Fig. 2). Lacunarity for each scale was computed by determining the lacunarity coefficient, which was calculated as the square of the coefficient of variation for each window, averaged across all windows. The lacunarity index, which characterizes the entire volume across scales, was derived from the slope of the log-log plot of lacunarity versus inverted window size [11, 12]. For various MRI sequences, tumor lacunarity was assessed by incorporating voxel values as an additional dimension, thereby transforming the 3D voxel volume into a 4D surface, which enabled lacunarity measurement via FracND. Due to the substantial computational demands of 4D measurements, subsampling at a rate of 0.01 (1:100) was implemented to reduce processing time.

### Statistical analysis

Comparisons of neuropathological diagnoses and categorical clinical variables were conducted via Fisher’s exact test, whereas continuous variables, all demonstrating normal distribution, were analyzed using independent samples t-test. Multivariable logistic regression was initially applied to fractal parameters in isolation, subsequently incorporating clinical parameters into the analysis. Scaling of continuous variables was performed to range from 0 to 1. Maximization of the area under the receiver operating characteristic (ROC) curve was achieved through separate greedy exhaustive searches for fractal and clinical parameters. Confirmation of variable selection was obtained through Fisher’s exact test or independent samples t-test (*p* < 0.05). Bootstrapped sampling with *n* = 100 and replacement was implemented to minimize overfitting effects (Supplement 1). The scoring system developed was derived from the mean regression weights across bootstrapped samples, with continuous variable scaling factors incorporated into the logits. Statistical analyses were conducted using Python for independent samples t-tests with SciPy, logistic regression with Scikit Learn and Fisher’s exact tests with R software.

### Limitations

- 48 patients, with varied pathologies,
- subjectivity and potential inter-surgeon variability in labeling tumor consistency from operative reports,
- at least T1c and FLAIR are required,
- calculation of the lacunarity index without subsampling is time-consuming (approximately one week per patient); subsampling reduces this to ~ 20 min depending on tumor size (in our case, computing needs were met with a system containing 2 x Intel(R) Xeon(R) Platinum 8268 CPUs and 900 GB of RAM), and.
- limited scales for fractal analysis due to MRI resolution, leading to potential measurement uncertainty.

## Results

### Descriptive statistics

We identified 48 patients, 34 (70.8%) females and 14 (29.2%) males, who met the selection criteria. The median age of the population was 62 years. Median tumor volume was 36 cm^3^. Of our patients, 56.2% had WHO grade 1, 43.8% had WHO grade 2 meningioma and no grade 3 meningioma was found in this cohort. In terms of consistency, 43.75% of the tumors were soft and 43.75% were hard, and in 12.5%, data were mixed. Fischer’s test showed no significant difference in consistency between patients with different WHO grades (*p* = 0.53).

### Fractal properties

We measured fractal dimension on segmentations and the lacunarity index on the T1, T1c, T2 and FLAIR sequences, as a fractal property.

We investigated the independence of fractal dimension and lacunarity index using two-sample T-test. At 5% significance level, LI is independent of the type of MRI sequence used for measurement. In other words, we can describe tumors by two independent fractal properties: LI and FD.

### Predictive models

Logistic regression was chosen to set up a predictive tool. The metrics used for the evaluation were the results of the ROC analysis, AUC, accuracy, precision and recall. The feature set with the highest score was selected. In the case of a tie, preference was given to smaller feature sets. During the exhaustive search, fractal parameters were treated independently of all other features. First, we selected the optimal fractal parameters, and with this optimal choice, an exhaustive search was performed among the other features.

We observed that bootstrapping yielded more conservative results for accuracy, precision, and recall. However, compared to cross-validation, it significantly reduced the risk of model overfitting, especially due to the small sample size of 48.

### Prediction of WHO grade based on fractal parameters

**Fig. 3:**
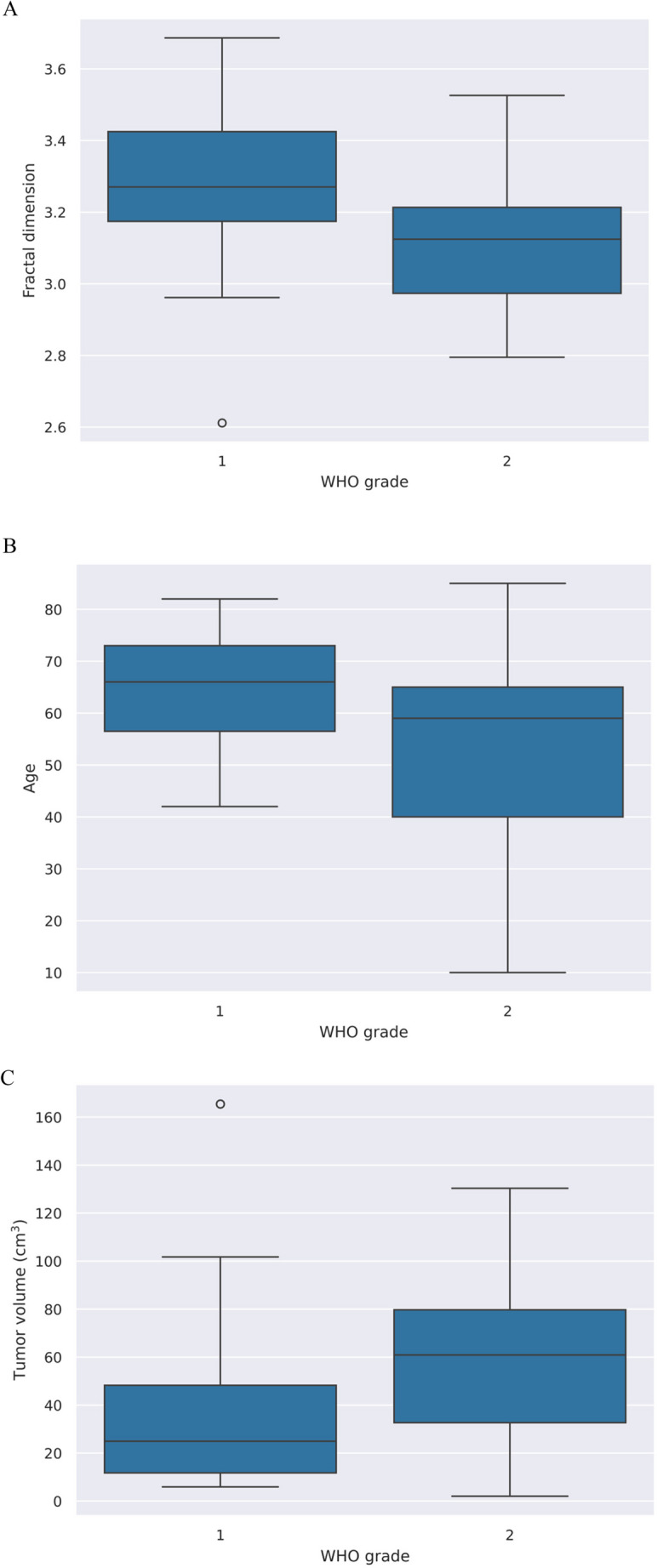
Boxplots comparing fractal dimension (**A**), age (**B**) and tumor volume (**C**). The thin outer edges represent the minimum and maximum, the bottom of the wide middle section represents the 25th percentile, the top the 75th percentile, and the middle line represents the group median (50th percentile). Outliers are indicated by white circles

For “continuous” variables (e.g., fractal dimension, age, tumor volume), independence was tested using an independent sample t-test. For categorical variables (e.g., homogeneity of contrast enhancement), we used Fisher’s test. The fractal dimension of tumors was significantly higher in the WHO grade 1 tumors (*p* = 0.009) (Fig. 3A). Patients were younger in the WHO grade 2 tumor group (*p* = 0.007) (Fig. 3B), and the higher tumor grade was significantly more often associated with larger tumor volume (*p* = 0.020) (Fig. 3C). Fischer’s test found a significant difference in contrast-enhancing property, with a greater chance of inhomogeneous enhancement at higher grade (*p* < 0.001) (OR = 10.785 (95% CI: 2.417–60.94).

**Fig. 4:**
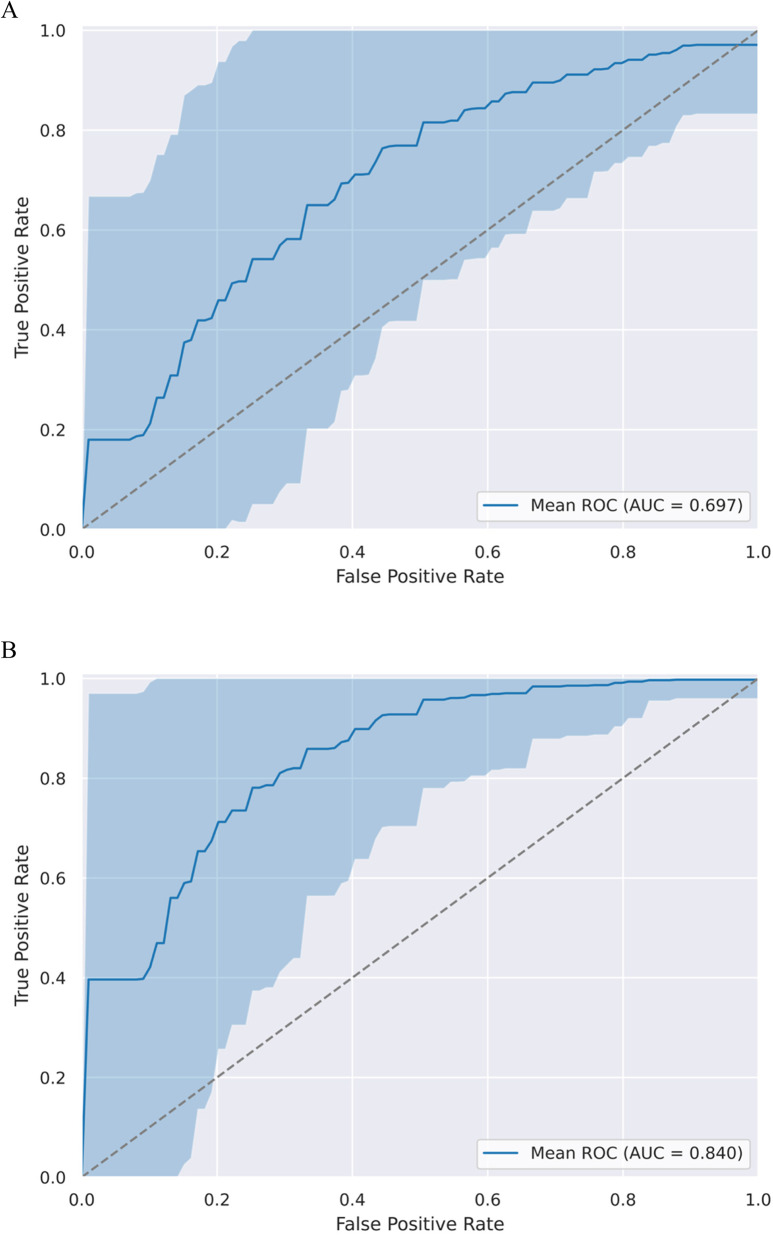
ROC analysis of the WHO grade prediction model. **A:** ROC curves (with CI intervals) and AUC values when only the fractal dimension was used to predict histopathology. **B:** ROC curves (with CI intervals) and AUC values when clinical parameters and conventional imaging were added to fractal properties to predict histopathology

When only the fractal parameter (FD) was used for prediction, the AUC value was 0.697 (95% CI: 0.490–0.952) (Fig. 4A). The inclusion of conventional imaging and clinical parameters substantially improved the predictive capability. When we used FD with age, tumor volume and contrast-enhancing property of tumors to predict pathological diagnosis, a mean of 0.841 AUC was found (95% CI: 0.625–1.000) (Fig. 4B**)**.

#### The scoring system for grade prediction

**Fig. 5:**
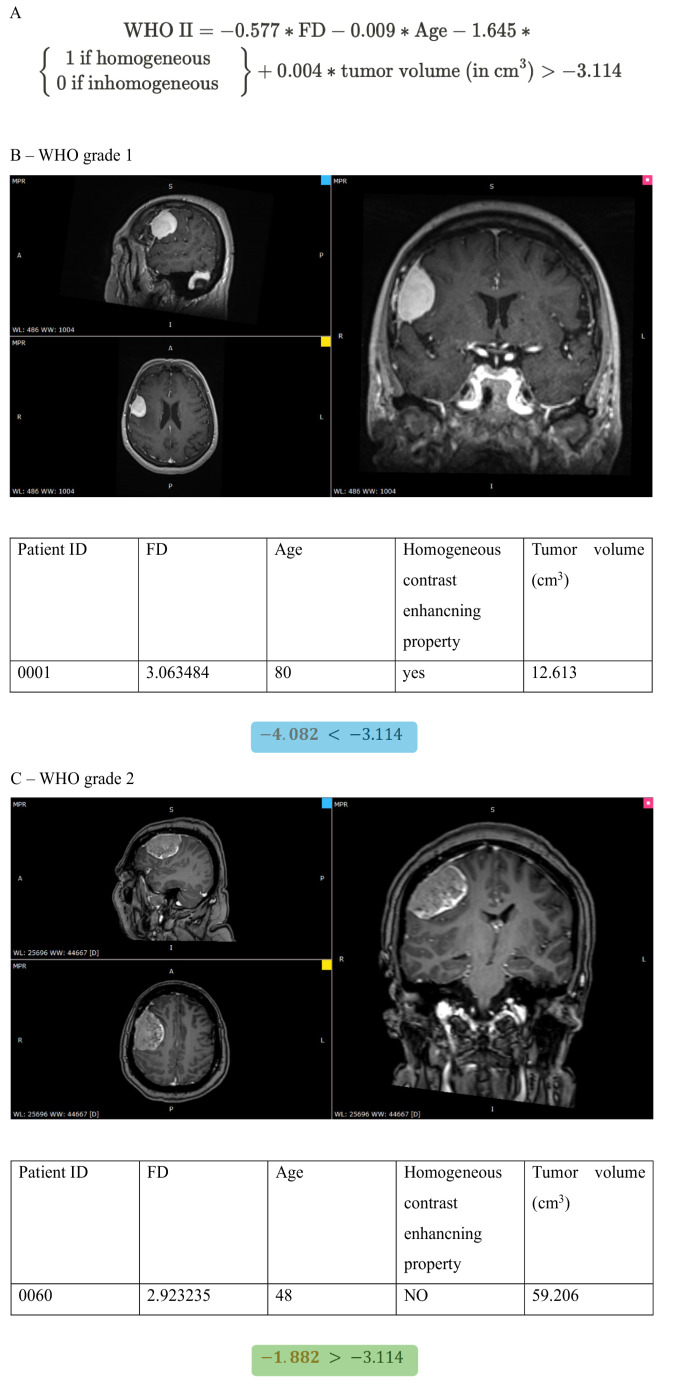
Demonstration of the use of our WHO grade prediction model in two cases. **A:** Our histopathology estimator formula. **B:** In a patient with CNS WHO grade 1 meningioma (T1-weighted contrast-enhanced axial, sagittal and coronal MRI images). (To calculate the total score, multiply the weights by the parameter value, and then sum them. If it is lower than the threshold for that class, the sample belongs to the “WHO grade 1 group”.) **C:** In a patient with CNS WHO grade 2 meningioma (T1-weighted contrast-enhanced axial, sagittal and coronal MRI images). (To calculate the total score, multiply the weights by the parameter value, and then sum them. If it is higher than the threshold for that class, the sample belongs to “WHO grade 2 group”.)

The predictive ability of each factor was weighted by the regression coefficients of the logistic regression analysis (Table 1). We created a prediction formula to calculate the total score by multiplying the weights with the corresponding parameter values and then summing up these products. The formula and the exact calculation method is illustrated in Fig. 5, where examples are provided for each tumor subtype.

**Table 1:** Weights of the predictor formula for WHO grades (derived from the regression coefficient of the logistic regression analysis)

Variable	Weight	OR (80% CI)	*p*-value
Fractal dimension	0.312	0.732 (0.573–0.955)	0.107
Age	−0.009	0.991 (0.986–0.998)	0.045
Homogeneous contrast enhancing property	−1.645	0.193 (0.109–0.328)	‹0.001
Tumor volume	0.004	1.004 (1.001–1.008)	0.151
Threshold	−3.114	undefined	undefined

### Prediction of tumor consistency based on fractal parameters

**Fig. 6:**
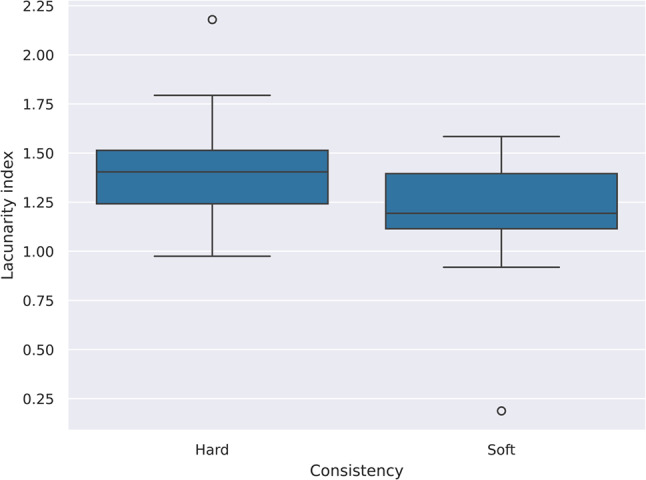
Boxplots comparing the lacunarity index between soft and hard consistency tumors

The LI of the tumors was significantly higher in hard consistency tumors (*p* = 0.025) (Fig. 6). Homogeneous contrast enhancement was more likely to occur in soft consistency tumors (OR = 3.411 (95% CI: 0.797–16.635)), but the difference was not significant (*p* = 0.111).

**Fig. 7:**
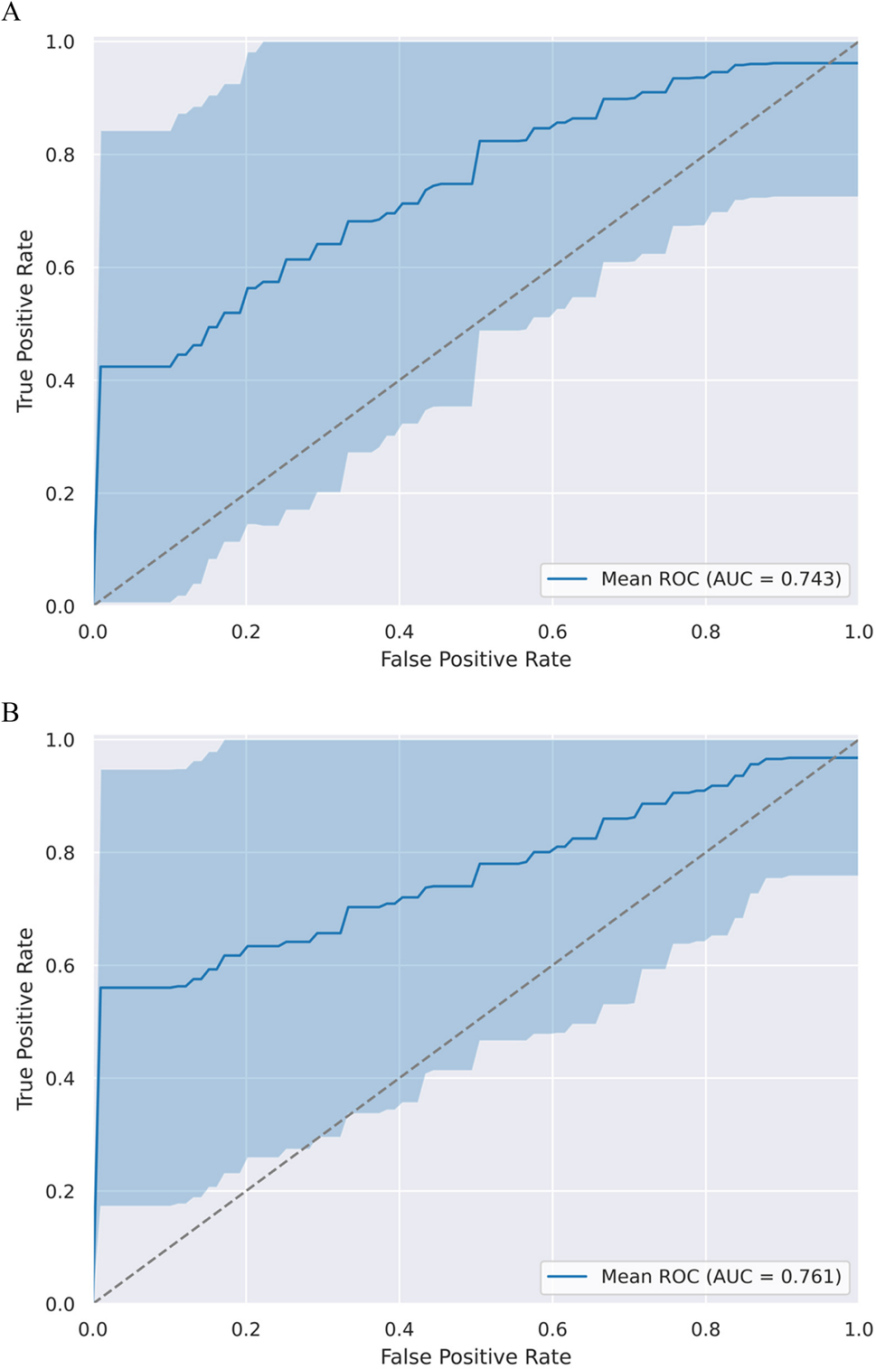
ROC analysis of the consistency prediction model. **A:** ROC curves (with CI intervals) and AUC values when only the lacunarity index was used to predict consistency. **B:** ROC curves (with CI intervals) and AUC values when conventional imaging was added to the fractal property to predict tumor consistency

When only fractal parameter (LI) was used for prediction, the AUC value was 0.745 (95% CI: 0.538–0.958) (Fig. 7A). The inclusion of conventional imaging parameters improved the predictive capability. When we used LI with the contrast-enhancing property of tumors to predict consistency, we found a mean AUC of 0.763 (95% CI: 0.518–1.000) (Figs. 7B**)**.

#### The scoring system for consistency prediction

**Fig. 8:**
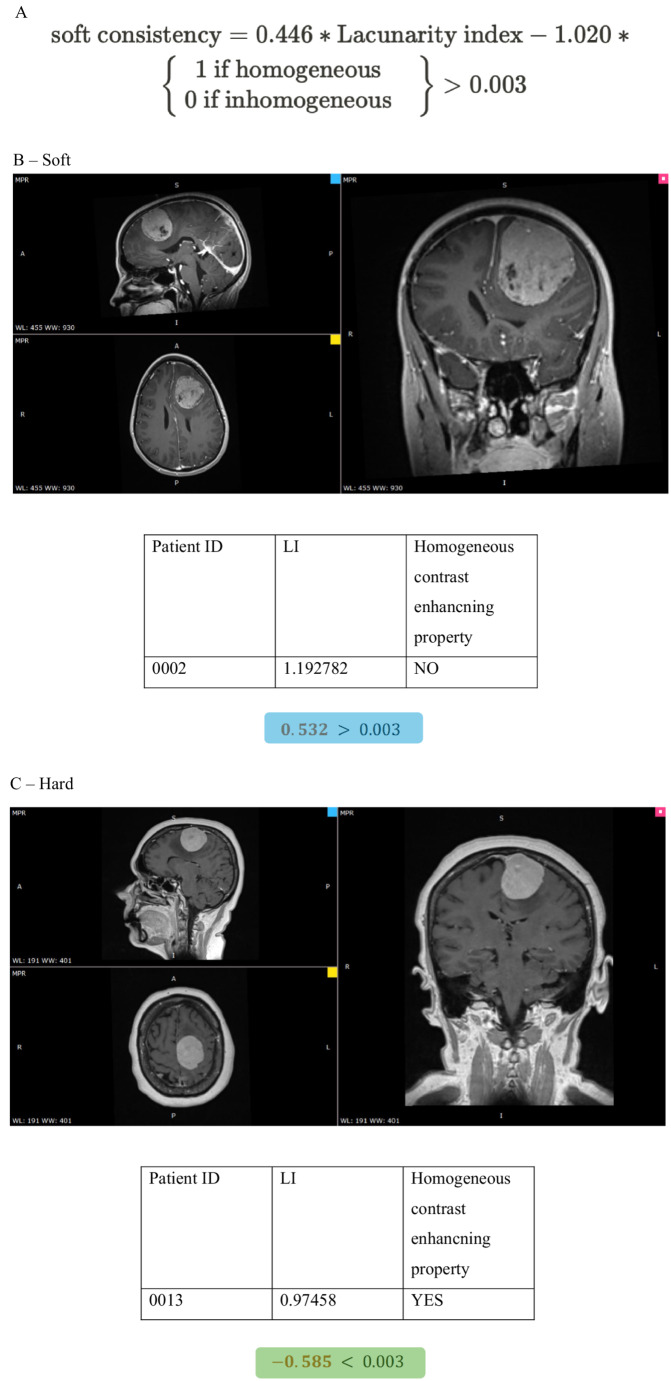
Demonstration of the use of our consistency prediction model in two cases. **A:** Our tumor consistency estimator formula. **B:** In a patient with soft consistency meningioma (T1-weighted contrast-enhanced axial, sagittal and coronal MRI images). (To calculate the total score, multiply the weights by the parameter value, and then sum them. If it is higher than the threshold for that class, the sample belongs to “soft” consistency group.) **C:** In a patient with hard consistency meningioma (T1-weighted contrast-enhanced axial, sagittal and coronal MRI images). (To calculate the total score, multiply the weights by the parameter value, and then sum them. If it is lower than the threshold for that class, the sample belongs to “hard” consistency group.)

The predictive ability of each factor was weighted by the regression coefficients of the logistic regression analysis (Table 2). We created a prediction formula to calculate the total score by multiplying the weights with the corresponding parameter values and then summing up these products. If it was higher than the threshold, the sample belonged to the “soft” consistency group, otherwise to the “hard” group. The formula and the exact calculation method are illustrated in Fig. 8, where examples are provided for two tumor subtypes.

**Table 2:** Weights of the predictor formula for consistency (derived from the regression coefficient of the logistic regression analysis)

Variable	Weight	OR (80% CI)	*p*-value
Lacunarity index	0.446	1.562 (1.265–1.943)	0.004
Homogeneous contrast enhancing property	−1.020	0.361 (0.194–0.645)	0.033
Threshold	0.003	undefined	undefined

##  Discussion

Meningiomas are the most common primary central nervous system tumors, accounting for 39.7% of cases [13]. Preoperative knowledge of the WHO grade can significantly influence the therapeutic plan for meningiomas, as their treatment ranges from close monitoring to radical tumor resection with or without postsurgical radiation therapy. A commonly employed strategy is surgical resection of tumors that show growth, edematous tissue reaction, or cause neurological symptoms [14]. The prognosis of meningiomas classified as CNS WHO grade 1 (low-grade) is significantly better than that of WHO grade 2/3 (high-grade) tumors [15], allowing for less aggressive treatment strategies. In contrast, patients with high-grade meningiomas may benefit from radical surgery (including dura and skull bone removal, combined with dura-skull reconstructive measures). With the recent spread of radiomic methods, significant research is being directed toward the non-invasive determination of the histological classification of meningiomas.

Traditional radiomics approaches predominantly analyze texture features at fixed spatial scales or examine local pixel neighborhoods to extract statistical measures of intensity distributions, whereas fractal geometry analysis provides a fundamentally different approach by capturing geometric complexity across multiple scales simultaneously. While conventional texture features primarily describe statistical relationships between neighboring pixels within predefined regions, fractal parameters provide a global characterization of the entire tumor’s structural complexity. Fractal dimension quantifies how irregular structures fill three-dimensional space and characterizes the self-similar, hierarchical patterns inherent in biological tissues, while lacunarity index measures the spatial distribution of gaps or heterogeneity within the tumor volume [6, 7]. This multi-scale geometric approach may be particularly relevant for pediatric brain tumors, where architectural complexity often reflects underlying biological processes such as cellular organization, vascular patterns, and growth dynamics that manifest at various spatial scales throughout the tumor volume.

The first research study investigating the relationship between fractal parameters determined from MRI images and the WHO grade of the tumor was published in 2016. Czyz et al. [16] determined FD values on different MRI slices, i.e., in 2 dimensions (2D), which showed a significant difference between low- and high-grade meningiomas. Their model considered several clinical and radiological parameters (absence of peritumoral edema, male sex, skull base location, age over 75, capsular contrast enhancement, non-homogeneous contrast enhancement) alongside FD, resulting in an AUC value of 0.83 in their cut-off point analysis for effectiveness determination. In their study, Park et al. [17] correlated the 3D FD and LI values of tumors with the WHO grade. Their model also included clinical and radiological parameters in addition to fractal properties (AUC = 0.82). These findings raise the question of whether 2D or 3D fractal parameters provide a more accurate WHO grade determination for meningiomas. Kim et al. [18] compared the usefulness of 2D and 3D fractal parameters. Their research showed that the model using 2D parameters (AUC = 0.690) significantly underperformed compared to the model using 3D parameters (AUC = 0.813). This outcome is consistent with the findings of Friconnet et al. [19], who used the 2D FD parameter to distinguish between WHO grade 1 and WHO grade 2/3 tumors with an AUC efficiency of 0.690. Fractal analysis according to the work of Won et al. [20] may also be suitable for distinguishing between WHO grade 2 tumors with wild-type and mutant TERT promoter regions, thus enabling not only the preoperative identification of histopathological results but also molecular markers.

Another significant factor influencing the complexity of surgery, and the extent of resection is tumor consistency [21]. Soft tumors can be removed by cutting and suction techniques, while more solid tumors, particularly skull base meningiomas, are more challenging. In such cases, additional surgical tools such as ultrasonic aspirators, electrophysiological monitoring, and intraoperative navigation are required [22]. Therefore, the development of a noninvasive preoperative method to predict tumor consistency is crucial. In the past, predictions were based on signal intensity from T2-weighted images or FLAIR images, but these methods had limited accuracy [23]. Radiomics has emerged as a promising approach for noninvasive, high-throughput analysis of tumor characteristics and has been applied to a variety of tumors, including pituitary adenomas and gliomas [24, 25]. However, there has been limited research on the use of radiomic features to predict meningioma consistency. Zhai et al. [2] aimed to develop a radiomics model for preoperative prediction of meningioma consistency in their latest paper. Their nomogram showed good sensitivity and specificity with AUC values of 0.861 and 0.960 in train and test cohorts, respectively, in predicting meningioma consistency.

In our study, we aimed to differentiate between soft and hard meningiomas and to predict the histological type based on fractal characteristics. To the best of our knowledge, studies exploring the use of fractal geometry analysis in predicting meningioma consistency have not yet been reported. When only fractal parameters were used for prediction, it was found that LI was able to separate the two subgroups (soft vs. hard). The AUC value was 0.745 (95% CI: 0.538–0.958) for consistency. When tumor homogeneity was added, these values changed to 0.763 (95% CI: 0.518–1.000). Using the same tools, we found that WHO grade could be predicted with an AUC value of 0.697 (95% CI: 0.490–0.952) using fractal dimension only. When we add more parameters such as age, tumor homogeneity and volume, this value increases to 0.841 (95% CI: 0.625–1.000).

The main limitation in evaluating predictions is the small sample size, which can result in substantial variability across individual bootstrap samples or cross-validation folds, making it easier for outliers to distort the metrics. Furthermore, excessive optimization for a particular random split may cause overfitting, leading to an overestimation of performance.

Fractal measurement with subsampling takes approximately 20 min per patient (system configuration: 2x Intel(R) Xeon(R) Platinum 8268 CP @2.90 GHz, 900 GB RAM), creating a significant bottleneck. Without subsampling, it would take about two weeks to achieve accurate measurements on the current hardware. This slowdown is mainly caused by the “curse of dimensionality,” where the number of boxes to evaluate increases polynomially with higher-dimensional objects, requiring a correspondingly larger number of CPU cores to sustain processing speed.

In conclusion, our results demonstrate that fractal dimension and lacunarity measurements are powerful predictors of noninvasive histopathological and tissue consistency information. When combined with structural imaging data, the multimodal feature set serves as an effective decision-support tool.

## Conclusion

Radiomics is an emerging method that provides additional information by extracting different quantitative metrics from medical images such as size, shape, texture, signal intensity and fractal features of the lesion.

Fractal geometry offers a powerful tool to characterize the intricacies of irregular and rough shapes inherent in these tumors. Fractal dimension serves as a concise metric, capturing the complexity of the shape of an object. It thus allows meaningful comparisons between different patient cohorts. To the best of our knowledge, this is the first report of a scoring system that combines fractal measures and clinical parameters to aid in preoperative preparations of meningioma surgeries.

Data from 48 patients showed that the two main tumor consistency types (soft and hard) were significantly distinct in terms of fractal dimension and FLAIR lacunarity property. The same tools were also used to differentiate between histological groups. A scoring system was constructed by combining fractal measures with conventional imaging parameters. The scores can aid in better preoperative diagnosis while leaving the decision at the surgeon, thus supporting surgical planning and prognosis estimation. These results suggest that fractal analysis using only routine imaging diagnostics can fine-tune the treatment of meningiomas and make personalized therapeutic decisions.

## Electronic supplementary material

Below is the link to the electronic supplementary material.

ESM 1 (DOCX 122 KB)

## Data Availability

No datasets were generated or analysed during the current study.

## Abbreviations

3D: 3 Dimensions
AUC: Area under the ROC curve
CI: Confidence interval
CNS: Central nervous system
CPU: Central processing unit
CUSA: Cavitron ultrasonic surgical aspirator
DICOM: Digital imaging and communications in medicine
FD: Fractal dimension
FLAIR: Fluid attenuated inversion recovery
GB: Gigabyte
LI: Lacunarity index
MRI: Magnetic resonance imaging
OR: Odds ratio
p: Probability
RAM: Random access memory
ROC: Receiver operating characteristic
T1c: T1 contrast enhancement
TERT: Telomerase reverse transcriptase
vs: Versus
WHO: World Health Organization
