# Supplementary material for: Consistency and grade prediction of intracranial meningiomas based on fractal geometry analysis
Source: Neurosurg Rev. 2025 Aug 14;48(1):598. doi: 10.1007/s10143-025-03737-1 (PMC12350593; doi:10.1007/s10143-025-03737-1)
Supplement: Supplementary file 1 — (DOCX 122 KB) [file 10143_2025_3737_MOESM1_ESM.docx]

# 1. Distribution of scores among the bootstrap samples (n = 100)


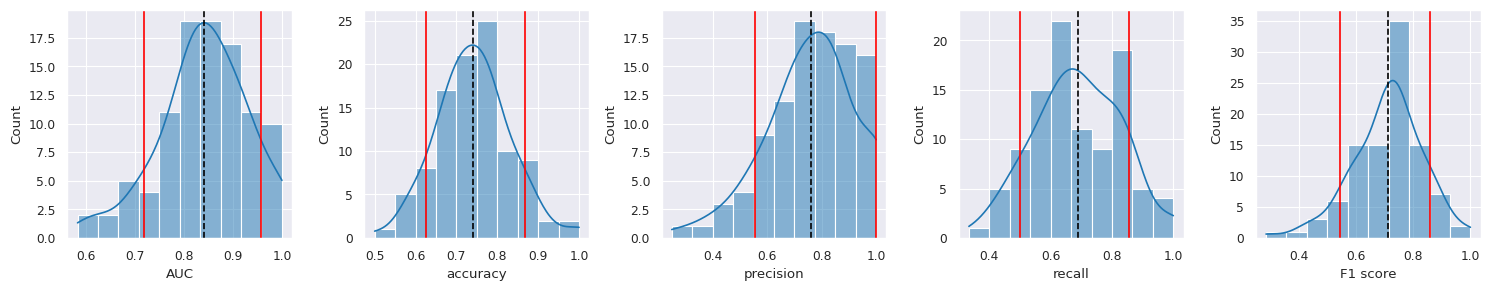


**Figure 1.:** Distribution of model metrics calculated on the out-of-boostrap samples of the logistic regression using FD, age, tumor volume and contrast-enhancing property to predict WHO grade. Solid blue line indicates the KDE of the distribution, red lines mark the 95% CI, dashed black line the mean.


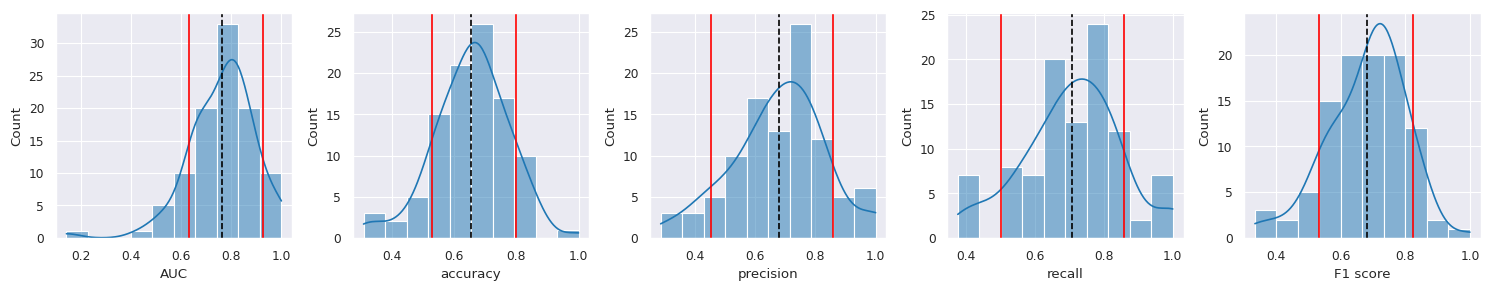


**Figure 2.:** Distribution of model metrics calculated on the out-of-boostrap samples of the logistic regression using LI with the contrast-enhancing property of tumors to predict consistency. Solid blue line indicates the KDE of the distribution, red lines mark the 95% CI, dashed black line the mean.
